# Prosthetic Joint Infection in Mega-Arthroplasty Following Shoulder, Hip and Knee Malignancy—A Prospective Follow-Up Study

**DOI:** 10.3390/life12122134

**Published:** 2022-12-17

**Authors:** Thilo Khakzad, Daniel Karczewski, Leonard Thielscher, Konstantin Reiter, Silvan Wittenberg, Alp Paksoy, Anne Flörcken, Daniel Rau, Sven Märdian

**Affiliations:** 1 Center for Musculoskeletal Surgery, Department of Orthopedics, Charité—Universitätsmedizin Berlin, Freie Universität Berlin and Humboldt-Universitat zu Berlin, Charitéplatz 1, D-10117 Berlin, Germany; 2 Department of Hematology, Oncology and Tumor Immunology, Charité—Universitätsmedizin Berlin, Freie Universitat Berlin and Humboldt-Universitat zu Berlin, D-10117 Berlin, Germany; 3 German Cancer Consortium (DKTK), Partner Site Berlin, German Cancer Research Center (DKFZ), Charité—Universitätsmedizin Berlin, D-69120 Heidelberg, Germany

**Keywords:** PJI, mega-prostheses, tumor arthroplasty, shoulder, hip, knee, comparison

## Abstract

Introduction: The risk of prosthetic joint infection (PJI) in mega-prosthesis for malignancy is increased compared with non-tumor cases. While several studies describe PJI in tumor-related arthroplasty, prospective studies comparing infection characteristics among different joints are limited. The present study analyzes mega-arthroplasty for hip, knee, and shoulder malignancy and compares the epidemiology, diagnosis, microbe spectrum, treatments, and outcomes between the different entities. Methods: The retrospective inclusion criteria were as follows: (1) mega-arthroplasty (2) in the hip, knee, or shoulder joint and a total femur arthroplasty (3) following a malignant bone tumor or metastasis (4) between 1996 and 2019. All included patients were prospectively followed and invited for a renewed hospital examination, and their PJI characteristics (if identified) were analyzed using both retrospective as well as newly gained prospective data. A PJI was defined according to the Infectious Disease Society of America (IDSA) and re-infection was defined according to the modified Delphi Consensus criteria. Results: In total, 83 cases of tumor mega-arthroplasty at a mean follow-up of 3.9 years could be included (32 knee, 30 hip, and 19 shoulder cases and 2 cases of total femur arthroplasty). In total, 14 PJIs were identified, with chondrosarcoma in 6 and osteosarcoma in 3 being the leading tumor entities. Knee arthroplasty demonstrated a significantly higher rate of PJI (*p* = 0.027) compared with hips (28.1% vs. 6.7%), while no significant difference could be found between the knee and shoulder (10.5%) (*p* = 0.134) or among shoulder and hip cases (*p* = 0.631). The average time of PJI following primary implantation was 141.4 months in knee patients, 64.6 in hip patients, and 8.2 months in shoulder patients. Age at the time of the primary PJI, as well as the time of the first PJI, did not show significant differences among the groups. Thirteen of the fourteen patients with PJI had a primary bone tumor. Statistical analysis showed a significant difference in the disadvantage of primary bone tumors (*p* = 0.11). While the overall cancer-related mortality in the knee PJI group (10%) was low, it was 50% in the hip and 100% in the shoulder group. Conclusion: The risk of PJI in knee tumor arthroplasty is significantly increased compared with hips, while cancer-related mortality is significantly higher in hip PJI cases. At the same time, mega-prostheses appear to be associated with a higher risk of infection due to a primary bone tumor compared with metastases. The study confirms existing knowledge concerning PJI in tumor arthroplasty, while, being one of the few studies to compare three different joints concerning PJI characteristics.

## 1. Introduction

While the prognosis of malignant bone tumors has significantly improved in the last 20 years due to new surgical and radio-chemotherapy-based approaches, the risk of prosthetic joint infection (PJI) in the field of mega-prosthesis following resection for tumors remains high [1]. The introduction of mega-prostheses as a limb-preserving therapy has significantly reduced the number of amputations required [2]. 

Many of the patients, especially those with primary bone tumors, have a high functional demand due to their young age. Even if the functional outcome of patients with mega-prostheses appears good overall, mega-prostheses have an extremely high complication rate of up to 80%, depending on the length of observation. 

The risk of PJI increases due to immunosuppression (chemotherapy, local radiation), large anatomic dead space, loss of soft tissue structures, longer operation times, and an increased tumor arthroplasty surface [3,4,5,6,7]. Reported infection rates vary between 10–20% and are thus more than ten times higher than arthroplasty for a degenerative disorder [7,8]. At the same time, the literature shows a difference in the infection rates between different joints [9]. The infection rates of shoulder prostheses after tumor resection are significantly lower and vary from 3.5% to 12% [5,10]. The different infection rates of the different joints can probably be explained by different soft tissue coverage, but this is still not fully understood [6]. The variation in the reported infection rates is, therefore, also due to a research desideratum. 

Interdisciplinary concepts, as well as the introduction of antiseptic surface coatings such as silver coating, have been able to reduce the risk of PJI [4,10,11]. 

Although being a severe and potentially deadly complication in immuno-incompetent patients, studies comparing PJIs in mega-prostheses for malignancy among joints (hip, knee, shoulder) are limited. 

Due to the high number of different secondary tumors in the form of bone metastases, it is often difficult to compare entities with regard to their susceptibility to infection after the implantation of a mega-prosthesis. We have made an attempt to distinguish between primary and secondary tumors.

Therefore, this study aimed to compare PJIs between different joints, including the epidemiology, tumor entities, symptoms, diagnosis, treatments, and outcomes. The present study results might allow for a more precise prognosis concerning prosthesis survival and mortality based on the involved joint type. 

## 2. Methods

### 2.1. Treatment Algorithm 

The present study was performed at a level I high care center providing treatment for approximately 6 million inhabitants. Treatment was provided by an interdisciplinary team of specialized orthopedic tumor surgeons, oncologists, infectiologists, and pathologists. Perioperative antibiotic prophylaxis in native tumor arthroplasty was provided by weight-based ampicillin/sulbactam (usually 3 × 3 g i.v.). In cases of known allergies, clindamycin, a licosami-antibiotic, was used instead of cephalosporin. At our center, we usually use antiseptic solutions containing polyhexanide, and we leave them in the surgical site for several minutes after surgical debridement. Then, the area is rinsed with 20 L of 0.9% NaCl with the help of a jet lavage. 

PJI treatment was based on the algorithm-based approach of Zimmerli and Trampuz et al. [12,13]. This PJI algorithm can be summarized as follows:

- Calculated antibiotic treatment in PJI was provided by ampicillin/sulbactam (3 × 3 g i.v.). The antibiotic therapy was combined with vancomycin (2 × 1 g i.v.) in septic patients with Methicillin-resistant Staphylococcus aureus (MRSA), multiple prior operations, or suspected low-grade infections. As soon as bacterial susceptibility could be identified, treatment was adjusted based on determined resistances and liver and renal function. Overall, antibiotic therapy in PJI was provided for at least 12 weeks and oralized based on the procedure and identified microbe.
- Debridement with antibiotics and implant retention (DAIR) was performed in acute infections (onset < 30 days), terminally ill patients, or as an attempt to achieve a prosthesis salvage procedure in surgically complex tumor cases. Following this, two weeks of i.v. antibiotics without antibiofilm activity and ten weeks of oral antibiotics with antibiofilm activity were administered after DAIR.
- In chronic cases (onset > 30 days), complete implant removal was performed [14]. A one-stage exchange was used in cases without known multi-resistant microbes, intact soft tissue envelopes without fistula, or multiple prior revisions. In all other cases, two- and three-stage exchanges were used. The latter was performed in failed previous PJI treatment attempts or cases involving microbes resistant to biofilm-active antimicrobials. Following a one-stage exchange, two weeks of i.v. antibiotics without antibiofilm activity were followed by ten weeks of oral antibiotics with antibiofilm activity. The two-stage exchange included the following protocol: prosthesis removal, two weeks of i.v. antibiotics without antibiofilm activity, four weeks of oral antibiotics without antibiofilm activity, re-arthroplasty, one week of i.v. antibiotics without antibiofilm activity, and five weeks of oral antibiotics with antibiofilm activity.

### 2.2. Inclusion and Exclusion Criteria 

In the first step, the following patients were retrospectively included in the study: (1) mega-arthroplasty implantation (2) in the hip, knee, or shoulder joint as well a total femur arthroplasty, (3) following a malignant bone tumor or metastasis (4) between 1996 and 2019. Exclusion criteria included (1) non-mega-arthroplasty, (2) implantation for non-malignant tumors, and (3) patients lost to follow-up.

### 2.3. Follow-Up 

All included patients were prospectively followed up via phone, postal service, or mail and invited to a renewed hospital examination if the last available internal medical records indicated that the patients were still alive (prospective follow-up study). The follow-up examination consisted of a brief history, a check for clinical signs of infection or tumor recurrence, and an X-ray. A board-certified orthopedic surgeon performed the physical examination. All patients with symptoms of infection, tumor recurrence, or further medical issues were subject to additional diagnostics and treatment as indicated and preferred by the patient. Patients who could not be contacted or refused to undergo a follow-up examination were excluded from the study. In patients who died in the interim, the date of death was considered the last follow-up. The local university’s ethics committee provided ethical approval (EA1/048/22), and the study was performed according to the Declaration of Helsinki. 

### 2.4. Analyzed Parameters 

All patients who underwent the follow-up examination or had official death reports (study group) were then checked for PJI events since primary mega-prosthesis implantation using clinical reports and the newly gained prospective data. PJI events between knee, shoulder, and hip tumor arthroplasty were compared concerning infection and demographic characteristics (number, age, score of the American Society of Anesthesiologists (ASA score), Charlson comorbidity index (CCI), tumor type, time of PJI, microbe spectrum, surgical treatment, antibiotics, leukocytes, histopathology, CrP, treatment strategies, and outcome). 

### 2.5. Definitions 

A PJI was defined according to the Infectious Diseases Society of America (IDSA) criteria [15] and considered present if one of the following criteria were fulfilled: (1) sinus tract to the prosthesis or periprosthetic purulence, (2) acute inflammation on periprosthetic histopathology, (3) microbial detection (one high-grade or ≥ 2 low-grade positive cultures), or (4) overall clinical judgment. In addition, the following were defined as infection-free using the modified Delphi Consensus criteria [16]: (1) absence of wound healing delay, sinus tract, or drainage; (2) no subsequent surgical intervention for infection; and (3) no infection-related mortality.

### 2.6. Statistics 

Descriptive statistics were reported as numbers (percentage) or mean (standard deviation). Calculations were performed using the SPSS version 25 software (SPSS Inc., Chicago, IL, USA). The Chi-square test was used for categorical variables and the t-test and Mann–Whitney U test for continuous variables. The level of significance was set to *p* < 0.05.

## 3. Results

In total, 83 cases of tumor mega-arthroplasty at a mean follow-up of 3.9 years could be included in the study (32 knee, 30 hip, and 19 shoulder cases) (Table 1). The follow-up of knee patients (5.7 years) was longer than the ones for the shoulder (2.5 years) and hip patients (3.2 years), while no significant difference between the groups was noted. Patients following knee tumor arthroplasty (49.7 years) demonstrated a younger age than hip patients (60.3 years) and shoulder patients (52.3 years) at the time of primary implantation.

There were no local or systemic signs of acute infection in any of the 83 patients examined. Likewise, there was no sign of loosening or similar in any of the radiographs taken. A total of 14 PJI events (16.8%) following arthroplasty for tumors were identified in the study. One PJI case included a total femur arthroplasty implanted for a myxofibrosarcoma that involved the entire femur (Table 2, case 10). As the infection had no clear primary focus (knee or hip), it was considered a separate type and was not calculated in the statistical calculations of the group comparison. 

Initial primary tumors in the PJI group included chondrosarcoma in six; osteosarcoma in three; Ewing sarcoma in two; and synovial-sarcoma, myxofibrosarcoma, and metastasis of a renal cell carcinoma in one case each. In total, 47 of the 83 patients had primary bone tumors, and 36 had secondary bone tumors in the form of metastases. Thirteen of the fourteen patients with PJI had a primary bone tumor. Statistical analysis showed a significant difference in the disadvantage of primary bone tumors (*p* = 0.11).

PJI microbes included Streptococci in four; coagulase-negative Staphylococci (CNS) in four; culture-negative cases in three; Enterococcus faecalis in two; and Staphylococcus aureus, Candida albicans, Klebsiella oxytoca, Escherichia coli, and Corynebacterium sp. in one case each.

**Figure 1:**
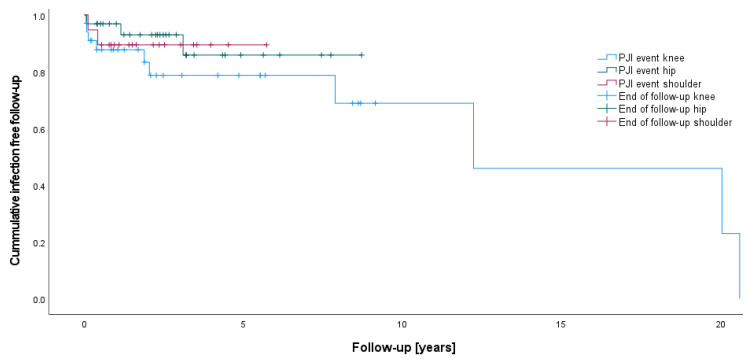
PJI events following knee, hip, and shoulder mega-arthroplasty for bone tumors.

Patients undergoing knee tumor arthroplasty demonstrated a significantly higher rate of PJI (*p* = 0.027) than hip patients (28.1 vs. 6.7%). In comparison, no significant difference could be shown between knee and shoulder (10.5%) (*p* = 0.134) or among shoulder and hip cases (*p* = 0.631) (Table 2, Figure 1). The average time of PJI following the primary implantation for bone tumor resection was 141.4 (SD ± 199.7months) in knee patients, 64.6 (SD ± 42.0 months) in hip patients, and 8.2 (SD ± 6.6 months) months in shoulder patients. Age at the primary PJI and time of first PJI did not show significant differences among the groups.

The analysis of the silver coating of the implants revealed no significant differences in the overall cohort. However, when comparing the subgroups, 9 of 32 knee joints were infected, with 21 being silver-coated. In the group of infections, only three were silver-coated, reaching statistical significance (*p* = 0.016). However, no significant differences could be detected at the hip or shoulder (Table 3).

Regarding the operating time, we compared the mean values of the PJI and non-PJI in the total cohort and the individual joint groups. Again, there was no significant difference (Table 3). To compare the comorbidities as influencing factors, we determined the CCI of the patients. There was a significantly higher CCI value in the PJI group (*p* = 0.008).

In all PJI cases, C-reactive protein (CrP) was increased with a mean of 70.2 mg (±80.9 mg) (>5 mg/L) directly before surgical treatment for PJI. However, no statistically significant difference between groups could be shown. Leukocytes were increased in 4 of 10 cases (>10 /nL), the mean was 9.0 (±2.6 /nL), and intraoperative histopathology demonstrated signs of infection in 9 of the 14 cases (Table 2).

All patients were treated with a combined antibiotic and surgical regimen. The most commonly used antibiotics included Ampicillin/Sulbactam in 9; Ciprofloxacin, a fluoroquinolone, and Vancomycin, a glycopeptide antibiotic, in 4 patients each; and Rifampicin, an asamycin antibiotic, in 3 of the 14 cases. DAIR and the removal of mobile parts were the treatments of choice in 5 of the 14 cases. Two of the five DAIR treatment attempts underwent prior isolated debridement before exchanging mobile parts and, in one of the five cases, additional debridement directly following DAIR. In addition, isolated debridement without an exchange of parts was performed in four cases, complete one-stage exchange of the prosthesis in two patients (resection arthroplasty with permanent arthrodesis), and three-stage exchange in one case each. Re-infection following initial surgical treatment for PJI was noted in four of the nine knee cases, while no re-infections were present in the hip or shoulder cases.

## 4. Discussion

While several descriptive reviews characterize PJI in tumor-related arthroplasty, limited original articles are present to date. In addition, there are limited prospective follow-up studies and studies comparing different joints within the group of original articles [17]. 

The present prospective cohort study identified mega-prostheses in the knee after malignancy as a risk factor for PJI compared with hip, but not shoulder, arthroplasty. Similar results were also described by Allison et al. The authors were able to show that prosthetic reconstructions of the hip (6.1% infection rate) demonstrate a significantly lower infection rate compared with knee arthroplasty in bone tumors (20.5%) (*p* < 0.001) [18]. Jeys et al. reported 1.240 patients at a mean follow-up of 5.8 years and a total infection rate of 11%. In their study, the tibial (23%) and pelvic sites (22.9%) of the tumor locations were identified as risk factors for PJI (<0.05). In contrast, the authors reported no significantly increased risk of PJI in shoulder cases [19]. Shehadeh et al. analyzed 232 patients treated with arthroplasty for malignant bone tumors at a minimum follow-up of 5 years and different localizations (knee, hip, shoulder, scapula). While identifying a total of 27 infections, PJI characteristics were not sub-analyzed for each joint [20]. Wouthuyzen-Bakker et al. analyzed 21 patients with chronic PJI (hip, knee, shoulder) who could not undergo revision surgery due to secondary diseases and were treated with antibiotic-suppressive therapy. In the authors’ cohort, treatment success was 90% in cases with a standard prosthesis (n = 11) compared with 50% with a tumor prosthesis (n = 10) [21]. However, the shoulder PJI identified by the authors was not part of the tumor arthroplasty group. 

The exact reason for the different outcomes between knee and hip tumor arthroplasty remains unknown. Karczewski et al. speculated that the higher risk of infection in knee arthroplasty might be due to less soft tissue around the joint than at the hip [22]. On the other hand, it might be due to the fewer surrounding muscles at the knee compared with the hip. Higher re-infection rates following treatment for knee PJI are also well-known in non-tumor arthroplasty [23,24]. However, further research identifying this discrepancy seems necessary. 

In addition to the location of the prosthesis, the tumor type might influence the rate of PJI. The infection itself is associated with a favorable prognosis concerning tumor recurrence [25]. This, in turn, might demonstrate that a higher rate of infections might be identified in less aggressive tumors. In the present study, PJI was only identified in one metastasis case, while the vast majority were caused in patients with osteosarcoma or chondrosarcoma. Our data suggest that patients with primary bone tumors have a higher risk of PJI. The reasons could be the subsequent aggressive systemic therapy and the possibly larger surgical resection. The localization of the knee may also be an influencing factor. Here, most patients had primary bone tumors, so the known susceptibility of the knee joint to infection distorts the result here. Likewise, the heterogeneity of secondary tumors with equally diverse post-treatments may permit an inadequate comparison here. However, survival bias should be critically reviewed in different studies when analyzing PJI rates in tumor arthroplasty based on underlying tumor type.

A significant risk factor for PJI is the presence of comorbidities. In this study, the patients with PJI were significantly sicker than the cohort measured by the CCI, so this must be included as a significant factor in the interpretation of the results.

The two-stage revision strategy remains an effective method for the treatment of PJI [26]. However, given the often-complex fixation of mega-arthroplasty in the bone, including cementation, a complete exchange remains challenging. Removing tumor arthroplasty can cause severe complications, including cement emboly, fracture, and cardiovascular risks. Therefore, it is often not performed as a first-line strategy in these cases but instead used as a last option to avoid septic shock or following repeated treatment failures. This is especially the case in terminally ill patients with limited life expectancy or severe secondary diseases (e.g., immunosuppression following chemotherapy), where isolated debridement or the exchange of mobile parts is preferred as a “minimally invasive salvage attempt”. In the present study, 9 of the 14 cases were treated with this approach, combined with adequate antimicrobial treatment. Re-infection was noted in three of these nine patients, as opposed to one re-infection in the remaining six cases, underscoring that implant retention is a reasonable strategy in selected patients with a PJI of a mega-prosthesis. 

Given the often-devastating consequences of PJI, infection prevention is of high importance in the first place. Therefore, in concordance with existing studies, our cohort used a first-generation cephalosporin as an antibiotic prophylaxis. While not evaluated in the present study, extended antibiotic prophylaxis [27,28] and the silver coating of the prosthesis surface [29] have demonstrated promising early results in the current literature. 

The identified microbe spectrum is atypical for PJIs [3]. While CNS, identified in four cases, is a typical cause of PJI, the most common microbe in PJI, Staphylococcus aureus, was only identified in one case. In contrast, Streptococci in four cases and culture-negative cases in four cases are atypical for PJI. Among the remaining cases, difficult-to-treat microbes were identified in one case (Candida albicans) [30]. High rates of Streptococci might represent an increased risk of hematogenous infection in mega-prostheses [31]. While the limited number of only 14 PJI cases is a limiting factor, an atypical microbe spectrum might contribute to the poor infection prognosis in tumor arthroplasty. 

The limitations of the present study include the small number of cases—only 14 PJI cases out of a cohort of 83 cases—and heterogenous follow-ups among different groups. Nonetheless, the study allows for a comparison among three different joints concerning PJI characteristics at a mid-to-long-term follow-up (>3.9 years).

## 5. Conclusions

Our data show that the risk of PJI is significantly increased in primary bone tumors compared with secondary bone tumors. The comparison of the different joints showed that PJI is significantly increased in knee tumor arthroplasty compared with the hip, while cancer-related mortality is significantly higher in cases of PJI in hips. Likewise, the data show that PJI after tumor arthroplasty is associated with an unusual spectrum of pathogens and that a hematogenous route of infection appears to be more common, especially in long-term follow-ups. At the same time, the study showed that patients with primary bone tumors have a higher risk of periprosthetic infection than those with secondary tumors.

This study confirms the existing knowledge about PJI in tumor arthroplasty and is also one of the few studies comparing three different joints with respect to PJI characteristics.

**Illustrative case**

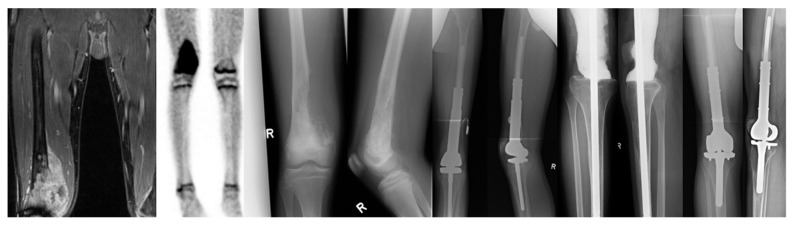



The above series of images shows the course of a patient who was 16 years old at the time of tumor diagnosis. He had an osteosarcoma of the right distal femur. Due to a positive growth prognosis, he initially received a growth prosthesis. This became infected 4 years later, so he received a complete removal with temporary arthrodesis after two DAIR attempts. The last picture is the one from the follow-up examination 9 years after re-implantation of a distal femur set.

## Figures and Tables

**Table 1:** PJI rates following tumor arthroplasty.

				*p*-Value		
	Knee (K)	Hip (H)	Shoulder (S)	K/H	K/S	H/S
Cases of tumor arthroplasty (n)	32	30	19	-	-	-
Age at time of primary implantation (years)	49.7 ± 23.3	60.3 ± 15.2	52.3 ± 18.6	0.128	0.876	0.129
Mean follow-up after implantation (years)	5.7 ± 6.4	3.2 ± 2.6	2.5 ± 1.4	0.490	0.284	0.682
PJI (n)	9 of 32	2 of 30	2 of 19	0.027	0.134	0.631
Infection rate (%)	28.1	6.7	10.5			
Age at time of primary PJI (years)	39.8 ± 26.5	74.0 ± 12.8	57.1 ± 16.7	0.218	0.327	0.667
Time to first PJI (months)	141.4 ± 199.7	64.6 ± 42.0	8.2 ± 6.6	0.727	0.145	0.333
CrP (mg/L)	80.2 ± 95.4	52.7 ± 65.7	47.6 ± 38.3	0.889	0.889	0.999
Leukocytes (/nL)	8.7 ± 3.2	9.6 ± 1.3	9.8 ± 0.2	0.999	0.711	0.999
Infectious histopathology (n)	5 of 9	1 of 2	1 of 2	0.637	0.637	0.999
Operation duration overall (min)	206.8 ± 46.8	226.6 ± 113.5	163.2 ± 31.5	0.643	0.004	0.234
Silver coating in overall (n)	21	20	16	0.930	0.001	0.175
Re-infection following initial treatment for PJI (n)	3 of 9	1 of 2	0	0.999	0.999	-
Cancer mortality (n)	0 of 9	1 of 2	2 of 2	0.026	0.001	0.248

**Table 2:** PJI characteristics.

Sex	Age at PJI	ASA	Primary Tumor	Time from PI to PJI	Microbe	CRP	Leukocytes	Histopathology	Antibiotics	Initial Surgery for PJI	Follow-Up Since Initial PJI	Outcome
Knee (localization of primary tumor is equivalent to arthroplasty replacement location; if both femur and tibia are affected, total knee arthroplasty was performed)												
Male	63	2	Chondrosarcoma, proximal tibia	4.6 months	Streptococcus agalacticae, Enterococcus faecalis	226 mg/L	10.5 /nL	Indifferent	Unacid	3x debridement; then DAIR	7.0 years	No re-infection; tumor survivor
Female	22	3	Osteosarcoma, distal femur	1.4 months	Staphylococcus aureus, Enterococcus faecalis	87.6 mg/L	5.9 /nL	Infection	Unacid, Ciprofloxacin, Rifampicin	2x debridement; then DAIR	7.0 years	No re-infection; tumor survivor
Male	25	2	Osteosarcoma, distal femur	95.9 months	Negative (fistula)	21.2 mg/L	7.8 /nL	n.a.	Unacid, Vancomycin	Debridement	5.9 years	Re-infection after 67 days (Staph. epidermidis; DAIR); then, infection freedom; tumor survivor
Male	65	2	Chondrosarcoma, proximal tibia	243.8 months	Streptococcus dysgalactiae	232.9 mg/L	14.4 /nL	Infection	Ampicillin/Sulbactam	DAIR	3.7 years	Re-infection after 21 days (Staph. Epidermidis; three-stage exchange) and after 664 days (Streptococcus agalacticae; DAIR); then, infection freedom; tumor survivor
Male	22	2	Osteosarcoma, distal femur	0.6 months	Negative (wound healing delay; purulence)	6 mg/L	4.3 /nL	n.a.	Unacid	Debridement and drain	1.3 years	No re-infection; tumor survivor
Female	85	2	Chondrosarcoma, distal femur	24.8 months	Enterococcus faecalis	28.2 mg/L	9.1 /nL	Infection	Unacid, Vancomycin	Debridement; then removal and permanent arthrodesis	2.1 years	No re-infection; tumor survivor
Male	53	1	Synovial-sarcoma, proximal tibia	148.8 months	Staphylococcus capitis, Corynebacterium sp.	27.3 mg/L	10.9 /nL	Infection	Cotrimoxazole, Tazobactam, Vancomycin, Ciprofloxacin, Daptomycin, Levofloxacin, Rifampicin	Three-stage exchange	6.6 years	Amputation for infection persistence after 414 days (Staphylococcus aureus, Staph. epidermidis); tumor survivor
Female	49	3	Ewing sarcoma, distal femur	250.6 months	No reports available (external surgery)						2.4 years	No re-infection; tumor survivor
Female	14	2	Ewing sarcoma, distal femur, proximal tibia	22.9 months	Streptococcus agalacticae	12.5 mg/L	6.6 /nL	Infection/Indifferent	Unacid, Vancomycin	DAIR	5.4 years	Re-infection after 95 days (culture negative, two-stage exchange with tibial component retention); then, infection freedom; tumor survivor
Total femur arthroplasty (tumor of the entire femur; removal of entire femur; femoral head, femur shaft, and total knee arthroplasty)												
Male	15	2	Myxofibrosarcoma, entire femur	0.9 months	*E. coli*, Staph. epidermidis	268.0 mg/L	5.8 /nL	Infection	Unacid, Ciprofloxacin	One-stage exchange	1.3 years	No re-infection; death by tumor
Hip (total hip arthroplasty was performed in both cases)												
Female	64	2	Chondrosarcoma, proximal femur	37.7 months	Staph. epidermidis	6.2 mg/L	8.8 /nL	Infection	Cotrimoxazole, Rifampicin	One-stage exchange	6.2 years	No re-infection; tumor survivor
Male	81	3	Chondrosarcoma, proximal femur	13.9 months	Staph. epidermidis, Candida albicans	99.1 mg/L	10.4 /nL	No information concerning infection	Cotrimoxazole, Voriconazole, Clindamycin	DAIR, then 2x debridement	0.8 years	No re-infection; tumor recurrence with hemipelvectomy after 57 days; non-tumor associated death
Shoulder (total shoulder arthroplasty was performed in both cases)												
Female	44	2	Chondrosarcoma, proximal humerus	5.1 months	Streptococcus sanguinis	20.5 mg/L	9.6 /nL	No infection	Unacid	Debridement	5.1 years	No re-infection; death by tumor
Male	68	3	Renal cell carcinoma metastasis, proximal humerus	1.4 months	Klebsiella oxytoca	74.6 mg/L	9.9 /nL	Infection	Ciprofloxacin, Unacid	2x Debridement	2.2 years	No re-infection; death by tumor

**Table 3:** Silver coating and operation time in group comparison.

	PJI	Non-PJI	*p*-Value
Number of events overall (n)	13	68	-
Silver coating overall (n)	6	51	0.178
Knee			
Number of events (n)	9	23	-
Operation duration (min)	185.5 ± 44.3	212.6 ± 46.8	0.309
Silver coating (n)	3	18	0.016
Hip			
Number of events (n)	2	28	-
Operation duration (min)	238.5 ± 111.7	225.7 ± 113.5	0.768
Silver coating (n)	2	18	0.301
Shoulder			
Number of events (n)	2	17	-
Operation duration (min)	147.5 ± 27.2	165.0 ± 30.6	0.655
Silver coating (n)	1	15	0.161

## Data Availability

The datasets generated and analyzed during the current study are available from the corresponding author upon reasonable request.
